# Cognition Is Associated With Peripheral Immune Molecules in Healthy Older Adults: A Cross-Sectional Study

**DOI:** 10.3389/fimmu.2020.02045

**Published:** 2020-09-02

**Authors:** Cláudia Serre-Miranda, Susana Roque, Nadine Correia Santos, Patrício Costa, Nuno Sousa, Joana Almeida Palha, Margarida Correia-Neves

**Affiliations:** ^1^Life and Health Sciences Research Institute, School of Medicine, University of Minho, Braga, Portugal; ^2^ICVS/3B’s – PT Government Associate Laboratory, Braga/Guimarães, Portugal; ^3^Clinical Academic Center – Braga, Braga, Portugal

**Keywords:** healthy aging, cognition, immune molecules, cytokines, chemokines

## Abstract

**Background:**

Cognition in the elderly is heterogeneous. Senescence of the immune system is increasingly considered as a potential player in cognitive performance. We explored here the interplay between cognitive performance and peripheral immune molecules in healthy older individuals.

**Methods:**

A cross-sectional study of clinically well characterized senior healthy individuals (120; 51–87 years old) previously clustered as “Good” and “Poor” performers based on established tests that evaluate memory and executive function. A plasma concentration of 30 immune molecules was assessed by multiplex analysis and correlated with parameters of cognitive performance.

**Results:**

Participants with worse cognitive performance (“Poor”) exhibited increased concentrations of IL-1β, IL-8, IL-13, and tumor necrosis factor (TNF) when compared to individuals with a better cognitive performance (“Good”). The cognitive dimensions memory and executive function, when considered separately, displayed a negative association with several immune molecules (IL-1β, IL-1RA, IL-6, IL-13, IP-10, and TNF with memory and only IL-1β with executive function), even controlling for age, sex, years of formal education, mood, and use of anti-inflammatory drugs. Regression analysis showed that several of these molecules (IL-1β, IL-6, IL-8, and IL-13) contribute to predicting whether an individual belongs to the “Good” or “Poor” cognitive performance group.

**Conclusion:**

These results strengthen the hypothesis that increased concentrations of peripheral immune molecules, like IL-1β, are associated with worse cognitive performance in senior healthy individuals. It further highlights that some poorly studied immune molecules should be considered in the context of cognitive aging, such as IL-13, here revealed as a new player in such interaction.

## Introduction

Understanding how peripheral immune mediators interact with the central nervous system (CNS) and influence cognition is of great relevance to provide clues on strategies to reduce or delay aging-associated cognitive decline in an increasingly aged population.

Several studies associate peripheral T lymphocytes and the cytokines they produce with cognitive performance, both in humans and in animal models (1, 2). Moreover, it was previously shown, in the same cohort of senior individuals studied here, that those displaying worse cognitive performance have a higher number of circulating effector memory (EM) CD4^+^ T cells (2). In fact, using regression models, EM CD4^+^ T cells were shown to contribute in predicting both memory and executive functions, controlling for age, sex, years of formal education, and mood (2). Interestingly, among T cells, EM CD4^+^ T cells are major cytokine producers in the periphery and possibly contribute to the increased inflammatory profile (also known as “inflammaging”) usually observed with aging (3). In accordance, an association between increased peripheral inflammatory profile with worse cognitive performances has been evidenced (4–6), with few studies addressing more than three immune molecules in cognitively healthy senior cohorts [IL-6 and C-reactive protein (CRP) are most commonly studied (4–6)]. Interestingly, studies in rodents revealed that bloodborne factors from old mice are able to impair spatial learning and memory as well as neurogenesis and synaptic plasticity in young animals (7). By contrast, blood from young animals is able to reverse age-related impairments (8). Even though the precise bloodborne factors that have the capacity to modulate and influence cognitive function are still under investigation, they likely relate to immune cells and/or immune mediators.

Hereupon, the aim of the present study was to perform an extensive analysis of peripheral immune molecules and explore their association with cognitive performance in a senior cognitively healthy population.

## Materials and Methods

### Participant Characterization

The sample of this study originates from a larger study in which a cohort representative of the older Portuguese population, in terms of sex and education, was selected from two districts in the North of Portugal (Guimarães and Vizela) (*n* = 1051). These individuals were extensively clinically and cognitively characterized (9). Cluster analysis identified neurocognitive/psychological performance patterns, and the two extreme groups were termed “Good” and “Poor” cognitive performers. For further characterization, a total sample of 120 subjects was selected, from both the “Good” and “Poor” cognitive performance groups, balanced for sex and age [sample size estimated assuming a two-tailed sample size, a medium effect size (*d* = 0.5), an alpha of 0.05, a statistical power of 0.8, and an equal sample size for each group]. The socio-demographic and clinical characterization of the participants is presented in Table 1. The recruitment and data acquisition took place between March 2012 and March 2013. Participants with incapacity and/or inability to attend the clinical and neuropsychological sessions, diagnosed with cognitive impairment or dementia and/or unable to understand informed consent, with disorders of the CNS or with overt thyroid pathology were not recruited. One participant was *a posteriori* excluded from the analysis due to impossibility of conducting blood collection.

**TABLE 1:** Socio-demographic, clinical, and neuropsychological characterization.

	**All participants**	**“Good” cognitive performers**	**“Poor” cognitive performers**	**“Good” vs “Poor”**
Total sample	119	64 (53.8%)	55 (46.2%)	n.s
**Sex**				
Male	63 (52.9%)	37 (57.8%)	26 (47.3%)	n.s
Female	56 (47.1%)	27 (42.2%)	29 (52.7%)	
**Age**				
Mean (range)	65.9 (51–87)	64.3 (51–82)	67.7 (52–87)	*
SD	8.4	8.3	8.2	
**School years**				
Median	4	4	4	***
0	3.4%	–	7.3%	
1–2	10.9%	6.3%	16.4%	
3–4	63%	56.3%	70.9%	
5–8	4.2%	6.3%	1.8%	
9–12	14.3%	23.4%	3.6%	
13+	4.2%	7.9%	–	
**Anti-inflammatory drugs**				
Yes	18 (15.1%)	8 (12.5%)	10 (18.2%)	n.s
**Immune to CMV**				
Yes	112 (94.1%)	60 (93.8%)	52 (94.5%)	n.s
**Neuropsychological evaluation**				
MEM (Mean; SD)	0.297; 1.209	1.245; 0.732	−0.807; 0.516	
GENEXC (Mean; SD)	0.031; 1.318	1.007; 0.923	−1.126; 0.557	
GDS (Mean; SD)	−0.023; 1.029	−0.343; 0.931	0.349; 1.021	

*p ≤ 0.05, ***p < 0.001, n.s.: non-significant, SD: standard deviation.

Considering the high prevalence of cytomegalovirus (CMV) infection in the Portuguese population and the impact of this chronic viral infection in the immune system, the presence of anti-CMV IgG was determined. Only 7 out of the 119 participants were non-immune to CMV; no correlations were observed between CMV antibody titers and the cognitive performance of the participants (data not shown).

### Cognitive Characterization

Trained psychologists evaluated the cognitive and mood profile of the participants as previously described (9). Briefly, the cognitive profile was established using a battery of neurocognitive and psychological tests selected to evaluate short-term verbal memory, verbal working memory, response inhibition/cognitive flexibility, verbal fluency, multiple trial verbal learning and memory, high-level information processing speed, global cognitive status, and mood. Using a principal component analysis, the neurocognitive/psychological test variables were grouped in three-dimensions: memory (MEM), general and executive function (GENEXEC), and mood (Geriatric Depression Scale—GDS). The cognitive groups, identified using cluster analysis, were classified as “Good” and “Poor” cognitive performers (9). Descriptive information with respect to scores for GDS, MEM, and GENEXEC of the total participants and of the “Good” and “Poor” cognitive performance groups is described in Table 1.

### Peripheral Immune Molecule Quantification

Blood was collected in EDTA tubes and processed for plasma collection and for standard hospital biochemical analysis. Plasma was obtained by gradient centrifugation using Histopaque 1077 (Sigma-Aldrich, United States) for 30 min, at room temperature, according to the manufacturer’s instructions, and stored at −80°C. Prior to use, plasma samples were centrifuged at 10,000 *g* for 10 min at 4°C to remove platelets and precipitates. Each participant was assigned a code, and all analyses were assessed blindly.

Cytokines, chemokines, and other immune molecules were quantified using multiplex magnetic bead-based immunoassays: Macrophage Inflammatory Protein-3β (MIP-3β)/Chemokine (C–C motif) ligand 19 (CCL19) using the MILLIPLEX MAP Human Cytokine/Chemokine Magnetic Bead Panel III—Immunology Multiplex Assay (Merck Millipore, United States); interleukin (IL)-1β, IL-1 receptor antagonist (RA), IL-2, IL-4, IL-5, IL-6, IL-7, IL-8, IL-9, IL-10, IL-12(p70), IL-13, IL-15, IL-17A, interferon (IFN)-γ, TNF, granulocyte-macrophage colony-stimulating factor (GM-CSF), G-CSF, interferon-γ-induced protein (IP)-10/C-X-C motif chemokine (CXCL)10, monocyte chemoattractant protein (MCP)-1/CCL2, MIP-1α/CCL3, MIP-1β/CCL4, RANTES/CCL5, and eotaxin/CCL11 using the Bio-Plex Human Cytokine 27-Plex Assay (Bio-Rad Laboratories, Lda., United States); and IL-33, IL-37, IFN-α, IFN-β, and IFN-ω using a Human ProcartaPlex Mix & Match (Invitrogen, United States). The plates were read using the Bio-Plex MAGPIX^TM^ Multiplex Reader and the data analyzed using the Bio-Plex Manager MP Software (both from Bio-Rad Laboratories, Lda., United States). The lower limit of quantification (LLOQ) and the percentage of detection for each analyte are listed in Supplementary Table 1. The inter-assay coefficient of variation was lower than 4.5%. Samples below LLOQ were extrapolated from the standard curve. The quantification of high-sensitivity C-reactive protein (hsCRP) was conducted at the certified Pathology Laboratory of Braga’s Hospital. Due to technical problems, three samples were not quantified for some of the immune molecules measured (sample size is presented in Supplementary Table 1).

### Statistical Analysis

Only analytes with a percentage of detection above 50% were considered for analysis. Outliers were defined by mean ± 3 standard deviations (SD) and excluded from the sample (Supplementary Table 1). Data for MEM, GENEXEC, and GDS were used in the analysis as *z*-scores, as previously determined (9). To evaluate normal distribution of the variables, skewness and kurtosis values were calculated and the approximate normal distribution was defined for variables with absolute values of skewness below 3 and of kurtosis below 8 (10). Considering the overall population, all analytes, except IL-6, followed a normal distribution. IL-6 concentration values were log10 transformed to comply with normality. Considering “Good” and “Poor” cognitive performance groups independently, IL-6 and G-CSF did not follow a normal distribution. Levene’s test was used to evaluate equality of variances.

To compare the peripheral concentration of immune molecules between “Good” and “Poor,” an independent-sample *t*-test was performed for variables with normal distribution and a Mann–Whitney U test for variables with non-normal distribution. Significance was considered for *p*-values equal or below 0.05. To quantify the strength of the differences, Cohen’s *d* was calculated as a measure of effect size (0.2 considered a small effect size, 0.5 a medium effect size, and 0.8 a large effect size) (11).

Linear regression analyses were performed to explore whether the various immune molecules (independently) were able to predict MEM or GENEXEC dimensions (dependent variables) and a binary logistic regression to explore whether the immune molecules were able predict to which cognitive group each individual belongs to (“Good” vs “Poor”), controlling for socio-demographic (age, sex, and school years) and clinical (GDS and anti-inflammatory drugs) variables. Multicollinearity between variables was assessed by the tolerance values (all the variables had a tolerance >0.5). The statistical procedures were performed in IBM SPSS Version 25 (IBM Corp., United States) and the graphs were designed using Prism7 (GraphPad Software, United States).

### Standard Protocol Approvals, Registrations, and Patient Consents

The study was performed in accordance with the Declaration of Helsinki and approved by ethics review boards (Hospital de Braga, Centro Hospitalar do Alto Ave, and Unidade Local de Sauìde do Alto Minho) and by the national data protection entity (Comissão Nacional de Proteção de Dados). All study goals and nature of the tests were explained, and informed signed consent was obtained from all participants.

## Results

### “Poor” and “Good” Cognitive Performers Present a Distinct Peripheral Immune Molecule Profile

**FIGURE 1:**
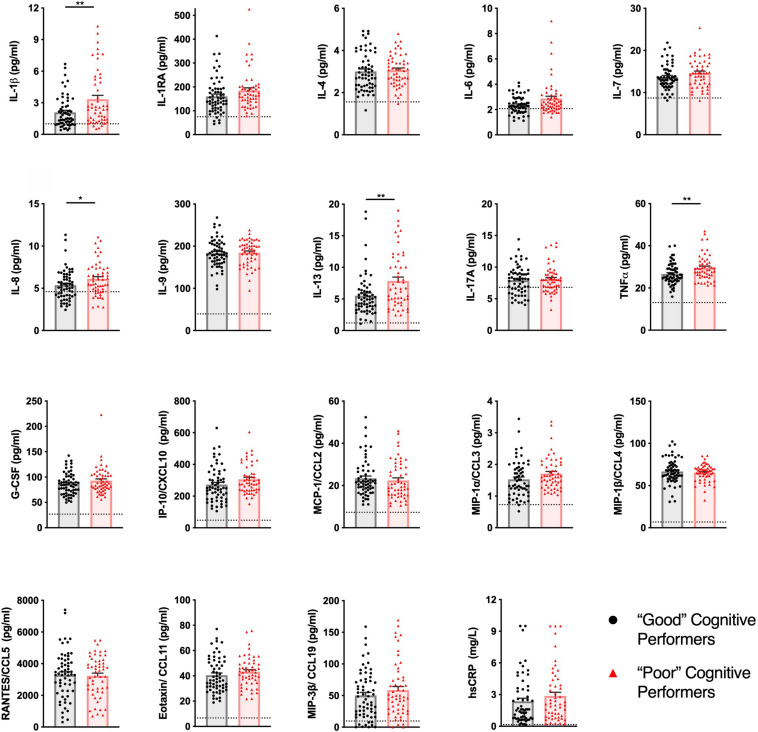
Healthy senior individuals with distinct cognitive performances present differences in the concentration of peripheral immune molecules. The profile of cytokines, chemokines, and other immune molecules in the plasma of “Good” (black circles) and “Poor” (red triangles) cognitive performers. Dashed lines represent LLOQ (lower limit of quantification), and values below LLOQ were extrapolated from the standard curve. Dots represent each participant, columns represent the mean of the group, and bars the standard error of the mean (*p < 0.05, **p < 0.01).

From the 31 analytes measured, 19 were detected in more than 50% of the samples (descriptive statistics and percentages of detection of each analyte for both groups are described in Supplementary Table 1). “Poor” cognitive performers had higher plasma concentrations of IL-1β, IL-8, IL-13, and TNF compared to “Good” cognitive performers (Figure 1 and detailed statistics in Supplementary Table 2). No differences were observed in the other analytes measured (Supplementary Table 2).

As an internal control, it was validated that all participants presented CRP levels below the limit associated with active inflammation/infection (10 mg/L) (12).

### IL-1β Is a Significant Predictor of Executive Function, Memory, and the Cognitive Clusters

Cognitive performance has been described to be directly, or indirectly, influenced by socio-demographic variables such as age, sex, and years of formal education (2, 9, 13) and by emotional health (14, 15). Considering also that the inflammatory profile can be influenced by age (a process known as “inflammaging”), sex, anti-inflammatory drugs, and mood (3, 16, 17), we next explored the association between cognitive function and the concentration of immune molecules controlling for these variables. Linear regression models were used to explain the variation of executive function (GENEXEC) and memory (MEM) dimension performances and binary logistic regression to discriminate between “Good” and “Poor” cognitive performers using each of the measured plasma molecules independently.

Regarding GENEXEC, only IL-1β, in addition to age, years of formal education, and mood, as previously reported (2), was found as a significant predictor (Table 2). The model explained approximately 46% (adjusted *R*^2^) of the variance observed in the GENEXEC dimension. Altogether, these observations indicate that, in the GENEXEC, “Good” performers have a lower peripheral concentration of IL-1β and, as shown previously, they are also younger, have more years of formal education, and have a better mood than “Poor” performers. To infer about the increment in the predictive power of the model due to the immune molecules, a hierarchical regression was performed. The first block was composed by variables needed to control for sex, age, school years, mood, and anti-inflammatory drugs. The second block was composed by the immune molecules, independently. Data on the concentration of IL-1β increased the predictive power of the model (*R*^2^ change) by 1.8%.

**TABLE 2:** Linear regression models to explain the variance of general and executive function (GENEXEC).

	**IL-1β**			
	***B***	**SE**	**β**	***t***
Sex^a^	–0.141	0.196	–0.054	–0.722
Age	–0.047	0.011	–0.302	−4.161***
School years	0.133	0.029	0.367	4.528***
GDS	–0.353	0.100	–0.279	−3.518**
Anti-inflammatory drugs	0.166	0.265	0.044	0.628
IL-1β	–0.084	0.043	–0.139	−1.945*
*F*(df1. df2)	15.635 (6; 106)***			
*R*^2^ (change)	0.489 (0.018)			
Adjusted *R*^2^	0.460			

SE, standard error; GDS, Geriatric Depression Scale. ^*a*^Reference category is male. *p ≤ 0.05, **p < 0.01, ***p < 0.001.

Regarding MEM, IL-1β, IL-1RA, IL-6, IL-13, IP-10, and TNF, in addition to age, mood, and number of years of formal education, were found as significant predictors (Table 3). Models (Table 3) (a model per molecule) explained between 29 and 32% (adjusted *R*^2^) of the variance observed in the MEM dimension. Altogether, these observations indicate that “Good” performers in the MEM dimension have a diminished peripheral concentration of some immune molecules. To infer about the increment due to the immune molecules in the predictive power of the models, a hierarchical regression was performed as described for GENEXEC. The levels of peripheral immune molecules increased the predictive power of the model (*R*^2^ change): IL-13 by 5.6%, followed by IL-1RA (3.2%), IL-1β and TNF (2.9%), IP-10 (2.8%), and IL-6 (2.7%). Body fat content can influence the inflammatory profile; however, in this cohort body mass index, whose values range from 19.5 to 38.2 kg per m^2^, did not influence the association between immune molecules and MEM and GENEXEC (data not shown).

**TABLE 3:** Linear regression models to explain the variance of memory (MEM).

	**IL-1β**				**IL-1RA**				**IL-6**			
	***B***	**SE**	**β**	***t***	**B**	**SE**	**β**	**t**	**B**	**SE**	**β**	**t**
Sex^a^	0.186	0.208	0.077	0.896	0.298	0.212	0.123	1.403	0.104	0.209	0.043	0.499
Age	–0.036	0.012	–0.253	−3.043**	–0.033	0.012	–0.226	−2.743**	–0.026	0.012	–0.178	−2.084*
School years	0.061	0.031	0.182	1.955*	0.070	0.031	0.209	2.262*	0.070	0.031	0.209	2.274*
GDS	–0.425	0.106	–0.364	−3.995***	–0.463	0.107	–0.390	−4.340***	–0.446	0.105	–0.380	−4.262***
Anti-inflammatory drugs	0.017	0.281	0.005	0.061	0.102	0.280	0.030	0.363	–0.009	0.283	–0.003	–0.031
IL-1β	–0.098	0.046	–0.175	−2.131*								
IL-1RA					–0.003	0.001	–0.186	−2.253*				
IL-6 (log10)									–1.470	0.702	–0.175	−2.096*
*F*(df1. df2)	8.595(6;106)***				8.821(6;107)***				8.914(6;108)***			
*R*^2^ (change)	0.327(0.029)				0.331(0.032)				0.331(0.027)			
Adjusted *R*^2^	0.289				0.293				0.294			

	**IL**-**13**				**IP**-**10**				**TNF**			
	**B**	**SE**	**β**	**t**	**B**	**SE**	**β**	**t**	**B**	**SE**	**β**	**t**

Sex^a^	0.184	0.202	0.076	0.911	0.173	0.208	0.071	0.831	0.167	0.207	0.068	0.807
Age	–0.033	0.012	–0.228	−2.793**	–0.023	0.013	–0.157	–1.792	–0.030	0.012	–0.206	−2.493*
School years	0.072	0.031	0.214	2.332*	0.067	0.031	0.200	2.158*	0.062	0.031	0.186	2.008*
GDS	–0.402	0.104	–0.343	−3.865***	–0.453	0.105	–0.387	−4.320***	–0.450	0.105	–0.381	−4.291***
Anti-inflammatory drugs	–0.040	0.277	–0.012	–0.144	–0.047	0.279	–0.014	–0.170	0.033	0.276	0.010	0.118
IL-13	–0.074	0.024	–0.244	−3.026**								
IP-10					–0.002	0.001	–0.180	−2.112*				
TNF									–0.038	0.017	–0.175	−2.174*
*F*(df1. df2)	9.745(6;105)***				8.575(6;107)***				9.079(6;107)***			
*R*^2^ (change)	0.358(0.056)				0.325(0,028)				0.337(0.029)			
Adjusted *R*^2^	0.321				0.287				0.300			

SE, standard error; GDS, Geriatric Depression Scale. ^*a*^Reference category is male. *p ≤ 0.05, **p < 0.01, ***p < 0.001.

The classification as “Good” or “Poor” performers was defined based on the individual scores in GENEXEC and MEM. To examine whether the immune molecules measured in plasma, together with sex, age, years of formal education, mood, and anti-inflammatory drugs, could account for the group delineation (“Good” vs “Poor”), a binary logistic regression was performed (Table 4). In addition to years of formal education and mood, IL-1β, IL-6, IL-8, and IL-13 (independently) were found as significant predictors. *R*^2^_Nagelkerke_ values ranged from 0.348 to 0.407, indicating that about 35 to 41% of the chance to belong to a particular cognitive group could be predicted by the model. The correct classifications of the subjects (% of total hit rates), based on the independent variables added in the model, ranged from 69 to 71%. To infer about the increment of the immune molecules in the predictive power of the models, the same rationale of the linear regression models was followed and a hierarchical regression performed. The levels of peripheral immune molecules increased the predictive power of the model (*R*^2^_Nagelkerke_ change): IL-13 (10.7%), followed by IL-1β (7.4%), IL-6 (4.4%), and IL-8 (3.5%).

**TABLE 4:** Binary logistic regression models to investigate the variables that discriminate between “Good” and “Poor” cognitive performers.

	**IL-1β**				**IL-6**			
	***B***	**SE**	**Wald**	**Exp(B)**	***B***	**SE**	**Wald**	**Exp(B)**
Sex^a^	–0.551	0.484	1.296	0.577	–0.452	0.481	0.884	0.636
Age	0.043	0.029	2.141	1.043	0.012	0.029	0.159	1.012
School years	–0.313	0.120	6.778**	0.732	–0.342	0.123	7.700**	0.710
GDS	0.506	0.240	4.452*	1.659	0.594	0.236	6.344*	1.811
Anti-inflammatory drugs	–0.345	0.625	0.305	0.708	–0.507	0.626	0.657	0.602
IL-1β	0.326	0.125	6.777**	1.385				
IL-6 (log10)					3.906	1.851	4.453*	49.708
χ^2^_ (df)_	36.653 (6)***				36.505 (6)***			
*R*^2^_Nagelkerke_ (change)	0.370 (0.074)				0.363 (0.044)			
Total hit rates (%)	69.000				68.700			

	**IL**-**8**				**IL**-**13**			
	***B***	**SE**	**Wald**	**Exp(B)**	***B***	**SE**	**Wald**	**Exp(B)**

Sex^a^	–0.558	0.475	1.384	0.572	–0.694	0.499	1.937	0.499
Age	0.030	0.028	1.139	1.031	0.039	0.029	1.847	1.040
School years	–0.333	0.125	7.095**	0.717	–0.371	0.122	9.178**	0.690
GDS	0.558	0.233	5.706*	1.747	0.498	0.243	4.213*	1.646
Anti-inflammatory drugs	–0.254	0.614	0.172	0.775	–0.173	0.634	0.074	0.841
IL-8	0.231	0.119	3.767*	1.260				
IL-13					0.225	0.071	9.926**	1.252
χ^2^_ (df)_	34.754 (6)***				40.648 (6)***			
*R*^2^_Nagelkerke_ (change)	0.348 (0.035)				0.407 (0.107)			
Total hit rates (%)	68.7				70.500			

SE, standard error; GDS, Geriatric Depression Scale. ^*a*^Reference category is male. *p ≤ 0.05, **p < 0.01, ***p < 0.001.

Of notice, inflammasome activation has been associated with cognitive symptoms in several human diseases (18) and with cognitive deficits in autoimmune experimental models (19). As such, we further investigated inflammasome activation as a possible mechanism behind the low-grade inflammation observed and the cognitive deterioration. To do so, the expression of five inflammasome activation markers (*nlrp3*, *aim2*, *pycard*, casp1, and *nlrc4*) (20) was analyzed in peripheral blood mononuclear cells from the same participants (Supplementary Methods and Supplementary Table 3). No differences were detected in the expression levels of these genes between “Good” and “Poor” cognitive performance groups (Supplementary Figure 1).

## Discussion

This study shows that, in a healthy senior cohort, the individuals with worse cognitive performance have higher plasma concentrations of IL-1β, IL-8, IL-13, and TNF. In addition, several immune molecules present a negative association with both memory (IL-1β, IL-1RA, IL-6, IL-13, IP-10, and TNF) and executive function (IL-1β), even controlling for age, sex, number of years of formal education, mood, and use of anti-inflammatory drugs. Interestingly, some of the immune molecules (IL-1β, IL-6, IL-8, and IL-13) are able to discriminate between “Good” or “Poor” cognitive performers.

Higher plasma concentrations of IL-1β negatively predict both memory and executive function and increase the odds of belonging to the “Poor” cognitive performance group in the regression models. These observations are in line with several previous studies, as follows: (i) IL-1β expression by activated monocytes predicts, negatively, cognitive performance in working memory in healthy senior individuals (21); (ii) IL-1β administration in rodents (both in the CNS and in the periphery) induces hippocampal-dependent memory deficits [reviewed in (22)]. However, some studies found no association or beneficial effect of IL-1β in cognitive function: (i) the serum concentration of IL-1β failed to demonstrate the association with several cognitive domains in healthy senior individuals (4); (ii) higher serum levels of IL-1β were associated with better semantic memory in older women even controlling for age, education, body mass index, and the presence of disease (23); (iii) blocking IL-1β signaling in rodents, both by administering IL-1RA (an IL-1b receptor antagonist) or by knocking out the IL-1b receptor, impaired memory and learning (24, 25); (iv) IL-1β administration in rodents had no effect or was beneficial on cognitive function [reviewed in (22)].

In addition to IL-1β, other immune molecules were found to associate with a specific cognitive domain: IL-1RA, IP-10, and TNF negatively associate with memory function; high concentrations of IL-8 increase the odds of belonging to the “Poor” performance group; and IL-6 and IL-13 both negatively associate with memory and increase the odds of belonging to the “Poor” performance group.

The role of IL-1RA on cognition has not been extensively addressed, and most of the available evidence originates from animal studies. To our knowledge, the present report is the first to describe a negative association between peripheral IL-1RA and memory in healthy older adults. Interestingly, the serum concentration of IL-1RA was also inversely correlated with cognitive function in a senior population with bipolar disorder (26); however, it may not necessarily affect cognition through the same mechanisms. Plus, intracerebral injection of IL-1RA, or its overexpression exclusively in the CNS, was shown to cause learning and memory impairments in various rodent models (24, 27). The overall evidence favors the hypothesis of a negative role of IL-1RA in the modulation of cognition. Most of the literature support that the pro-inflammatory profile, usually observed in senior individuals, is associated with a worse cognitive profile (4–6). Interestingly, IL-1RA has mainly an anti-inflammatory action [by blocking one of the IL-1β receptors (28)] and seems to be negatively associated with cognition.

IP-10, also known as IFNγ-IP 10 or CXCL10, is secreted by several cell populations in response to IFNγ. Here we observed that IP-10 concentration negatively associates with working memory performance after controlling for age and other confounders [as also shown by others (29)]. More so, IFNγ was below the detection limit for most individuals in our cohort, but, interestingly, IFNγ was identified as a negative regulator of cognitive functioning in rodents (30).

Regarding TNF, a negative association with memory is here shown. Another study in which TNF was part of a composite score of various immune biomarkers showed a negative association with processing speed, attention, and executive functioning, but not with memory (5). In addition, most reports in healthy human elders (mostly older than 70 years and based on cognitive assessment diverse from ours) found no association between peripheral TNF concentration and cognitive performance (4, 6, 31). Discordant findings on the role of TNF in cognition emerge as well from rodent studies (32–34). Nevertheless, it is possible that the role of TNF on cognition may be age-dependent. In accordance, McAfoose et al. (34) showed that TNF-/- mice perform worse than WT when they are young and better when they are old (34).

Interleukin-8, although poorly studied in the context of cognition, was one of the immune molecules that increased the odds to belong to the “Poor” cognitive performance group in the regression model, which is in accordance with other studies (4, 35). Still, in a large sample, another report showed no association between IL-8 and cognitive function in non-demented community-dwelling elderly individuals (6).

Interleukin-6 is one of the best-studied cytokines in aging. The vast majority of studies report a negative association between peripheral concentration of IL-6 and cognitive performance (6, 31, 36, 37), mainly with measures of executive function, and not of memory (6, 36, 37). In this study, we show no association between the concentration of IL-6 and executive function, but higher levels of IL-6 associate with worst memory and increase the odds of belonging to the “Poor” cognitive performance group. Of note, in several other studies it has also been shown to predict cognitive decline (31, 38, 39). The majority of the literature, including ours, associates an increase in peripheral IL-6 with worst memory function in seniors [although two studies reported no association (4, 40)].

To our knowledge, only one publication indicated that IL-13 deficiency in rodents significantly impairs working and reference memory (41). Here, we observed the opposite—IL-13 not only negatively associates with memory but also increases the odds of belonging to the “Poor” cognitive performance group. These results highlight the need to perform more studies using cohorts composed by healthy aging individuals and addressing less explored immune molecules like IL-13 and others.

The major study strengths are its extensive evaluation of peripheral immune molecules and the cohort being composed by extremely well-characterized healthy seniors; the comprehensive neuropsychological characterization assessing two distinct cognitive domains (memory and executive function); and the control for active inflammatory responses (through quantification of CRP) and for the use of anti-inflammatory drugs. Nonetheless, the cross-sectional design does not allow causal conclusions about the relationship between peripheral profile of immune molecules and age-associated cognitive decline.

A note on the inconsistencies observed between human studies is warranted. These may possibly occur due to differences in selection, characterization, and inclusion/exclusion criteria. For instance, the age ranges and the sample sizes vary greatly, as well as in the diversity in the cognitive evaluation tools and in the concentration range of the immune molecules measured. More so, not all studies check or control for possible active inflammatory processes or the intake of anti-inflammatory drugs (4, 23). CRP, an acute phase protein, is vastly studied in geriatric populations and has been associated with an increased risk for cerebrovascular disease, Alzheimer’s disease, and vascular dementia (42). Some studies described no association between levels of CRP and cognitive performance in healthy senior individuals (6, 40, 43) as we do here, while others demonstrate an association between higher levels of CRP and worst cognitive performance (31, 36, 44, 45). Interestingly, in those studies the CRP range is lower than in this study, indicating that other factors that vary between cohorts may mediate or dilute its impact on cognition.

## Conclusion

In summary, this study presents an extensive evaluation of peripheral immune molecules in a healthy senior population comprehensively characterized for cognitive performance, allowing a broader perspective on the association between the peripheral profile of immune molecules and cognitive performance. The data support the hypothesis that the peripheral profile of immune molecules at older ages is related with cognitive performance. Furthermore, it reinforces the pertinence to explore the possible interactions between cytokines in the modulation of cognitive function. It seems that an overall increased concentration of immune molecules, both pro- and anti-inflammatory, associates with stronger cognitive deterioration during aging. Additionally, a reasonable set of these studies, preferentially performed in distinct geographic regions, are necessary to feed secondary research, like meta-analysis, to be able to integrate the data from independent studies and clearly define the immune molecules that coherently associate with cognitive functions. Furthermore, the precise mechanisms behind the impact of peripheral immune mediators in the cognitive function are still unknown. Animal models are likely to shed some light on those mechanisms.

Understanding how the immune mediators interact with the CNS and influence cognition is of great relevance to provide clues on potential strategies to reduce or delay aging-associated cognitive decline in an increasingly aged population.

## Data Availability Statement

The raw data supporting the conclusions of this article will be made available by the authors, without undue reservation to any qualified investigator.

## Ethics Statement

The studies involving human participants were reviewed and approved by ethics review boards (Hospital de Braga, Centro Hospitalar do Alto Ave, and Unidade Local de Sauìde do Alto Minho) and by the national data protection entity (Comissão Nacional de Proteção de Dados). The patients/participants provided their written informed consent to participate in this study.

## Author Contributions

CS-M had a major role in acquisition of data, performed the statistical analysis of the data, interpreted the data, and wrote the first draft of the manuscript. SR and NS designed and conceptualized the study and revised the manuscript for intellectual content. NCS established the cohort and revised the manuscript for intellectual content. PC participated in establishment of the cohort, assessment, and statistic advisory. JP and MC-N designed and conceptualized the study, interpreted the data, and revised the manuscript for intellectual content. All authors contributed to the article and approved the submitted version.

## Conflict of Interest

The authors declare that the research was conducted in the absence of any commercial or financial relationships that could be construed as a potential conflict of interest.
