# Supplementary material for: Cognition Is Associated With Peripheral Immune Molecules in Healthy Older Adults: A Cross-Sectional Study
Source: Front Immunol. 2020 Sep 2;11:2045. doi: 10.3389/fimmu.2020.02045 (PMC7493640; doi:10.3389/fimmu.2020.02045)
Supplement: Supplementary file 1 [file Data_Sheet_1.docx]

**Supplementary Information**

**Cognition is associated with peripheral immune molecules in healthy older adults: a cross-sectional study**

Cláudia Serre-Miranda, MSc ^a,b^, Susana Roque , PhD ^a,b^, Nadine Correia Santos, PhD ^a,b,c^, Patrício Costa, PhD ^a,b,c^, Nuno Sousa, MD PhD ^a,bc^, Joana Almeida Palha, PhD ^a,b^, Margarida Correia-Neves, PhD ^a,b,*^

**^a^** Life and Health Sciences Research Institute (ICVS), School of Medicine, University of Minho, Braga, Portugal

**^b^** ICVS/3B’s - PT Government Associate Laboratory, Braga/Guimarães, Portugal

**^c^** Clinical Academic Center – Braga, Braga, Portugal

***Corresponding author:** Margarida Correia-Neves, Life and Health Sciences Research Institute, School of Medicine, University of Minho, Campus de Gualtar, 4710-057 Braga, Portugal.

E-mail: mcorreianeves@med.uminho.pt; telephone number: +351 253 604 807.

This document is composed by Supplementary Methods, Supplementary Table 1, 2 and 3 and Supplementary Figure 1

**Supplementary Methods**

**Quantitative real-time PCR analysis**

Total RNA was isolated from PBMCs (peripheral blood mononuclear cells), previously collected and stored in liquid nitrogen (plasma and cells were isolated from the same blood samples), using a PureLink® RNA Mini Kit (ThermoFisher Scientific) according to the manufacturer’s instructions. For cDNA synthesis 1µg of RNA was converted into cDNA using the iScript™ cDNA Synthesis Kit (Bio-Rad). qRT-PCR was performed in a CFX96 System (Bio-Rad) using the SsoFast™ EvaGreen® Supermix (Bio-Rad). A melting curve analysis was also carried out to verify the specificity of amplicons. The analysis was performed in the CFX Maestro™ Software and the ΔΔCt method was used to quantify the amount of mRNA level relative to housekeeping genes: *actin*, *b2m* and *gapdh*. All the housekeeping genes presented an average M value inferior to 0.5. The oligonucleotides used for qRT-PCR analysis are listed on Supplemental Table 3. To normalize for the 2 independent runs performed for each gene due the sample size, a z-score method was applied. Samples from the “Good” and “Poor” Cognitive Performance groups were distributed equally by the 2 runs. The inter-run CV (%) was below 10% for all genes, except for *casp1* (13%) and *nlrp3* (19%). To compare the relative expression of genes associated with inflammasome activation between “Good” and “Poor” cognitive performers, an independent-sample t-test was performed.

**Supplementary Table 1. Descriptive statistics of the immune molecules measured in plasma of “Good” and “Poor” cognitive performers.**

|  |  |  | **All Participants** | |  |  | **"Good" Cognitive Performers** | | | | |  |  | **"Poor" Cognitive Performers** | | | | |
| --- | --- | --- | --- | --- | --- | --- | --- | --- | --- | --- | --- | --- | --- | --- | --- | --- | --- | --- |
|  | **Molecules** | **LLOQ (pg/mL)** | **Outliers (n)** | **% Detection** |  | **N** | **% Detection** | **Mean** | **SD** | **Min.** | **Max.** |  | **N** | **% Detection** | **Mean** | **SD** | **Min.** | **Max.** |
| **Molecules with >50% samples above LLOQ** | IL-1β | 1.00 | 3 | 84% |  | 61 | 77% | 2.09 | 1.47 | 0.40 | 6.68 |  | 52 | 93% | 3.34 | 2.61 | 0.52 | 10.28 |
|  | IL-1RA | 74.84 | 2 | 96% |  | 65 | 92% | 153.00 | 80.25 | 0.00 | 413.20 |  | 55 | 100% | 174.98 | 90.04 | 0.00 | 525.88 |
|  | IL-4 | 1.56 | 2 | 98% |  | 61 | 98% | 3.01 | 0.91 | 1.16 | 4.92 |  | 53 | 98% | 3.06 | 0.73 | 1.48 | 4.80 |
|  | IL-6 | 2.07 | 1 | 75% |  | 62 | 72% | 2.37 | 0.66 | 1.12 | 4.12 |  | 53 | 78% | 2.86 | 1.40 | 1.40 | 9.00 |
|  | IL-7 | 8.71 | 4 | 98% |  | 61 | 98% | 13.67 | 2.95 | 8.12 | 21.84 |  | 51 | 98% | 14.64 | 3.38 | 8.12 | 25.36 |
|  | IL-8 | 4.59 | 1 | 72% |  | 61 | 66% | 5.37 | 1.79 | 2.44 | 11.32 |  | 54 | 78% | 6.12 | 1.98 | 2.76 | 11.08 |
|  | IL-9 | 39.33 | 1 | 100% |  | 61 | 100% | 183.43 | 33.13 | 98.08 | 267.88 |  | 54 | 100% | 184.57 | 29.99 | 95.16 | 238.16 |
|  | IL-13 | 1.20 | 4 | 99% |  | 60 | 98% | 5.51 | 3.34 | 1.08 | 18.80 |  | 52 | 100% | 7.85 | 4.37 | 2.44 | 19.04 |
|  | IL-17A | 6.80 | 2 | 72% |  | 61 | 69% | 7.94 | 2.29 | 4.04 | 14.40 |  | 53 | 76% | 8.08 | 2.17 | 3.28 | 13.88 |
|  | TNF | 13.07 | 2 | 100% |  | 61 | 100% | 26.64 | 5.01 | 15.88 | 40.04 |  | 53 | 100% | 29.58 | 6.08 | 21.24 | 46.96 |
|  | G-CSF | 26.75 | 1 | 100% |  | 61 | 100% | 86.26 | 21.72 | 49.84 | 142.44 |  | 54 | 100% | 92.73 | 26.56 | 55.84 | 223.28 |
|  | IP10/CXCL10 | 47.71 | 2 | 100% |  | 61 | 100% | 272.56 | 110.21 | 105.52 | 629.92 |  | 53 | 100% | 305.97 | 91.93 | 151.52 | 604.28 |
|  | MCP-1/CCL2 | 7.29 | 2 | 100% |  | 60 | 100% | 23.39 | 8.74 | 11.00 | 52.36 |  | 54 | 100% | 22.43 | 8.86 | 10.48 | 45.92 |
|  | MIP-1⍺/CCL3 | 0.73 | 1 | 99% |  | 61 | 98% | 1.53 | 0.57 | 0.52 | 3.44 |  | 54 | 100% | 1.71 | 0.51 | 1.04 | 3.36 |
|  | MIP-1β/CCL4 | 6.76 | 0 | 100% |  | 62 | 100% | 66.80 | 14.59 | 30.80 | 102.16 |  | 54 | 100% | 65.13 | 10.19 | 32.52 | 85.60 |
|  | RANTES/CCL5 | 12.63 | 0 | 100% |  | 62 | 100% | 3293.75 | 1495.24 | 309.12 | 7401.32 |  | 54 | 100% | 3226.14 | 1300.56 | 686.28 | 5484.64 |
|  | CCL11/Eotaxin | 6.68 | 2 | 100% |  | 61 | 100% | 40.51 | 13.88 | 18.92 | 77.04 |  | 53 | 100% | 43.19 | 11.96 | 21.72 | 75.64 |
|  | CCL19 | 9.67 | 1 | 89% |  | 63 | 86% | 50.25 | 37.64 | 0.00 | 158.93 |  | 55 | 93% | 58.45 | 43.20 | 0.00 | 169.20 |
|  | hsCRP | 1.60E+05 | 1 | 99% |  | 61 | 98% | 2.36E+06 | 2.31E+06 | 1.60E+05 | 9.50E+06 |  | 54 | 100% | 2.87E+06 | 2.54E+06 | 2.30E+05 | 9.50E+06 |
|  |  |  |  |  |  |  |  |  |  |  |  |  |  |  |  |  |  |  |
| **Molecules with <50% samples above LLOQ** | IL-2 | 4.64 | 1 | 8% |  | --- | --- | --- | --- | --- | --- |  | --- | --- | --- | --- | --- | --- |
|  | IL-5 | 14.79 | 2 | 13% |  | --- | --- | --- | --- | --- | --- |  | --- | --- | --- | --- | --- | --- |
|  | IL-10 | 6.75 | 2 | 16% |  | --- | --- | --- | --- | --- | --- |  | --- | --- | --- | --- | --- | --- |
|  | IL-12p70 | 4.88 | 1 | 8% |  | --- | --- | --- | --- | --- | --- |  | --- | --- | --- | --- | --- | --- |
|  | IL-15 | 141.47 | 1 | 5% |  | --- | --- | --- | --- | --- | --- |  | --- | --- | --- | --- | --- | --- |
|  | IL-33 | 22.79 | 1 | 0% |  | --- | --- | --- | --- | --- | --- |  | --- | --- | --- | --- | --- | --- |
|  | IL-37 | 4.20 | 1 | 29% |  | --- | --- | --- | --- | --- | --- |  | --- | --- | --- | --- | --- | --- |
|  | IFN-𝛾 | 7.08 | 1 | 7% |  | --- | --- | --- | --- | --- | --- |  | --- | --- | --- | --- | --- | --- |
|  | IFN-⍺ | 2.43 | 3 | 0% |  | --- | --- | --- | --- | --- | --- |  | --- | --- | --- | --- | --- | --- |
|  | IFN-β | 7.11 | 2 | 0% |  | --- | --- | --- | --- | --- | --- |  | --- | --- | --- | --- | --- | --- |
|  | IFN-ω | 5.11 | 1 | 31% |  | --- | --- | --- | --- | --- | --- |  | --- | --- | --- | --- | --- | --- |
|  | GM-CSF | 3.33 | 2 | 7% |  | --- | --- | --- | --- | --- | --- |  | --- | --- | --- | --- | --- | --- |

Abbreviations: LLOQ (Lower limit of quantification); N (sample size); SD (standard deviation).

**Supplementary Table 2. Detailed statistics on the comparison between the immune molecules measured in plasma of “Good” and “Poor” cognitive performers.**

| **Independent-sample t-test** | | | | |
| --- | --- | --- | --- | --- |
| **Molecules** | **t** | **df** | **p value** | **d** |
| **IL-1β** | -3.049 | 77.409 | **>0.01** | 0.590 |
| IL-1RA | -1.685 | 112 | 0.10 | --- |
| IL-4 | -0.374 | 112 | 0.71 | --- |
| IL-7 | -1.608 | 110 | 0.11 | --- |
| **IL-8** | -2.128 | 113 | **0.04** | 0.397 |
| IL-9 | -0.193 | 113 | 0.85 | --- |
| **IL-13** | -3.137 | 94.591 | **>0.01** | 0.602 |
| IL-17A | -0.342 | 112 | 0.73 | --- |
| **TNF** | -2.833 | 112 | **>0.01** | 0.348 |
| IP10/CXCL10 | -1.742 | 112 | 0.08 | --- |
| MCP-1/CCL2 | 0.584 | 112 | 0.56 | --- |
| MIP-1⍺/CCL3 | -1.762 | 113 | 0.08 | --- |
| MIP-1β/CCL4 | 0.721 | 109.089 | 0.47 | --- |
| RANTES/CCL5 | 0.258 | 114 | 0.80 | --- |
| CCL11/Eotaxin | -1.096 | 112 | 0.28 | --- |
| CCL19 | -1.103 | 116 | 0.27 | --- |
| hsCRP | -1.113 | 113 | 0.27 | --- |
|  |  |  |  |  |
| **Mann-Whitney U test** | | | | |
| **Molecules** | **U** | **Z** | **p value** |  |
| IL-6 | 1339.5 | -1.704 | 0.088 |  |
| G-CSF | 1430.5 | -1.213 | 0.225 |  |

Abbreviations: df (degrees of freedom); d (Cohen’s d).

**Supplementary Table 3. List of primers used for cDNA amplification.**

| **Genes** | **Forward primer** | **Reverse primer** |
| --- | --- | --- |
| *actin* | GCC GTC TTC CCC TCC ATC GTG | GGA GCC ACA CGA GCT CAT TGT AGA |
| *b2m* | AGC AGC ATC ATG GAG GTT TGA | TCA AAC ATG GAG ACA GCA CTC A |
| *gapdh* | CCT CCT GTT CGA CAG TCA G | CGA CCA AAT CCG TTG ACT C |
| *nlrp3* | AAG GGC CAT GGA CTA TTT CC | ACT CCA CCC GAT GAC AGT TC |
| *pycard (asc)* | AAG CCA GGC CTG CAC TTT AT | GGT ACT GCT CAT CCG TCA GG |
| *aim2* | CGT GCT GCA CCA AAA GTC TC | GGC AAA CAG CTT CTG AAA |
| *casp1* | CCA CAA TGG GCT CTG TTT TT | CAT CTG GCT GCT CAA ATG AA |
| *nlrc4* | AAC TCG AGC TTG GGT TG | TTC CCG CCA AAT TCA ACT GC |

**Supplementary Figure 1: No differences were found in the expression levels of inflammasome-related genes between “Good” and “Poor” cognitive performers.** The profile of normalized expression (relative to *actin*, *b2m* and *gapdh*) of 5 genes associated with the inflammasome activation in PBMCs collected from “Good” (black circles) and “Poor” (red triangles) cognitive performers. Standardized values are presented to account for inter-run variations. Dots represent each participant and lines the mean for each gene.
